# Understanding dementia in minority ethnic communities: The perspectives of key stakeholders interviewed as part of the IDEAL programme

**DOI:** 10.1177/14713012241272817

**Published:** 2024-08-17

**Authors:** Christina R. Victor, Eleanor van den Heuvel, Claire Pentecost, Catherine Quinn, Catherine Charlwood, Linda Clare

**Affiliations:** Department of Health Sciences, College of Health, Medicine and Life Sciences, 3890 Brunel University London, London, UK; Department of Health Sciences, College of Health, Medicine and Life Sciences, 3890 Brunel University London, London, UK; University of Exeter Medical School, 3286 University of Exeter, Exeter, UK; Centre for Applied Dementia Studies, Faculty of Health Studies, 1905 University of Bradford, Bradford, UK; Wolfson Centre for Applied Health Research, Bradford, UK; University of Exeter Medical School, 3286 University of Exeter, Exeter, UK; NIHR Applied Research Collaboration South-West Peninsula, Exeter, UK; University of Exeter Medical School, 3286 University of Exeter, Exeter, UK; NIHR Applied Research Collaboration South-West Peninsula, Exeter, UK

**Keywords:** ethnicity, understanding dementia, belonging, racism, responding to dementia, the migrant lifecourse

## Abstract

Future populations of older adults in the UK, those aged 65+, will demonstrate increased diversity in terms of their ethnic identity resultant from the ageing of the post-war migrants from India, Pakistan, Bangladesh, and the Caribbean. As a consequence, there will be an increase in the numbers of older adults from these communities living with age-related chronic diseases such as dementia. In response to these demographic changes, we need to develop a research, policy and practice agenda that is inclusive and provides evidence for the development of culturally diverse and effective models of service delivery. This requires engagement with three key stakeholder groups: (a) people with dementia; (b) their carers; and (c) the wider community. As part of the IDEAL research programme on living well with dementia, we undertook semi-structured interviews with twelve community leaders, defined as known and trusted individuals active in their respective communities, and six community members (two people living with dementia and four carers). We explored their understandings, experiences, and views of about dementia. Our analysis identified two overarching themes. The migrant lifecourse highlighted issues of not belonging, discrimination and racism. This framed our second theme, the cultural context of dementia, which addressed dementia knowledge and attitudes, service provision and service access, and how being part of a minority ethnic community made a difference to these experiences. Our study highlights how lifecourse experiences of negative hostile social and policy environments and services can be profound and long-lasting and provide a prism through which accessing dementia care is experienced. Our findings argue for the inclusion of diverse views and lifecourse experiences within the context of developing a dementia strategy for research, policy and practice that is appropriate for a multicultural and heterogenous society.

## Introduction

Future populations of older adults in the UK will be characterised by increasingly heterogeneous identities as illustrated by the ageing of the post-war migrants from India, Pakistan, Bangladesh, and the Caribbean. Current population projections suggest that by 2050 27% of those aged 65+ will be from ethnic minority groups compared with 5% in 2021 (Office for National Statistics, 2021). One consequence of this will be the increasing numbers of older adults from these communities living with age-related diseases such as dementia. Therefore, we need a research agenda that is inclusive and provides evidence for the development of culturally diverse effective models of service delivery (Alzheimer’s Society, 2021). This requires engagement with three key stakeholder groups: (a) people with dementia; (b) their carers; and (c) the wider community.

There is some evidence examining the understanding and experiences of dementia among Asian, Caribbean, and African populations in the UK from the perspectives of people with dementia and their carers (Brown et al., 2021; Hossain et al., 2020; Kenning et al., 2017; Lasrado et al., 2021; Roche et al., 2021). Although community leaders are seen as key sources of support and information about all aspects of dementia, including research, within their respective communities (Alzheimer Europe, 2018; Forbat, 2003; Waheed et al., 2020) few studies have actively sought their views (Baghirathan et al., 2020). The British IDEAL research programme, a longitudinal cohort study of people with dementia and family carers recruited from memory clinics (Clare et al., 2014; Silarova et al., 2018), included a workstream focussing on the experiences of Black and South Asian (Indian, Pakistani, and Bangladeshi) communities. To establish the context within which people from these groups experience ageing and dementia, we explored the understandings, experiences and views of community leaders, people with dementia and caregivers from these minority ethnic groups.

## Methods

The work reported here was conducted in London and Southeast England. Given the nature of our study we used an exploratory descriptive qualitative design (Hunter et al., 2019). Our study population consisted of three key stakeholder groups from Black African/Caribbean and South Asian heritage: community leaders, defined as known and trusted individuals active in their respective communities, people with dementia, and caregivers of people with dementia. Participants self-defined their alignment with these criteria. We used a pragmatic recruitment strategy including introductions to key stakeholders by an independent health and care consultant, Brunel University research engagement networks, established links with the communities of West London, and snowballing. Participants were interviewed either in person or by phone at their home, place of work or other location of their choice. All interviews were in English and undertaken by the researcher (EvdH).

We used semi-structured interviews to facilitate discussions with interviewees about their life in the UK, knowledge of and attitudes towards dementia, how they perceived dementia as being experienced and understood by their community, and how they thought being part of a minority ethnic community made a difference to these experiences. Following discussion with potential participants, we used the term ‘memory problems’ rather than dementia in the interviews, which were conducted in English between May 2018 and December 2019 and lasted approximately 45 minutes each. Interviews were professionally transcribed verbatim, and data managed using NVivo Version 12. The study was approved by the College of Health and Life Sciences Research Ethics Committee at Brunel University London (reference 10598-LR-Mar/2018-12350-2 and 11745-MHR-Jul/2018-13456-2).

Inductive thematic analysis followed the six -stage framework of Braun and Clarke (2022). Transcripts were coded independently by EvdH and CV with discussion to develop the overall themes. Feedback on our analysis was provided by 15 participants via telephone rather than in-person because of COVID restrictions.

## Findings

We recruited 18 participants: 12 community leaders, referred to as CL, and six community members: two people living with dementia and four carers, referred to as CM (Table 1). Ages of those interviewed ranged from 27 to 89 years and 13 were women.

**Table 1.** Characteristics of participants.

ID	Role	Sex	Faith	Age bracket	Ethnicity (country)
CL1SA	Muslim Chaplain at University	M	Islam	50–60	South Asian
CL2BC	Day centre manager and community organiser	F	Christian	50–60	Black Caribbean
CL3SA	Hindu scholar and community leader	M	Hindu	60–70	South Asian (Indian)
CL4SA	Director of a charity for Asian people with disabilities with day centre and home care services	F	None	30–40	South Asian
CL5BC	Retired social worker and volunteer for a mental health charity started to support people from the Caribbean community	F	Christian	60–70	Black Caribbean
CL6BA	Residential care home manager in Southwark	F	Christian	50–60	Black African
CL7SA	Young leader in Sikh temple	M	Sikh	20–30	South Asian
CL8SA	Committee member and preacher at Sikh temple	M	Sikh	55–65	South Asian
CL9SA	Former social worker who started her own day centre for Asian older people	F	Sikh	20–30	South Asian (Afghan)
CL10BC	Retired nurse active in the community	F	Christian	70–80	Black Caribbean
CL11SA	Lead for the BME dementia service of a mental health charity	F	Sikh	30–40	South Asian
CL12BC	Director of a charity supporting Caribbean families Joint interview with CM6BC	F	Christian	70–80	Black Caribbean (Barbados)
CM1SA	Living with alcohol-related dementia	M	Christian	50–60	South Asian (Indian)
CM2BC	Retired professional living with dementia	M	Atheist	80–90	Black Caribbean (Guyana)
CM3BC	Carer whose mother had died 4 years previously	F	Christian	50–60	Black Caribbean (Grenada)
CM4BC	Carer not living with her mother	F	None	50–60	Black Caribbean (St Lucia)
CM5BC	Retired nurse with concerns about her memory	F	Christian	60–70	Black Caribbean
CM6BC	Carer whose mother had died the previous year. Joint interview with CL12	F	Christian	40–50	Black Caribbean

Key

Community Leader = CL.

Community Member = CM

SA = South Asian.

BC = Black Caribbean.

BA = Black African.

Our analysis identified two overarching themes. The first related to the migrant lifecourse. Within this theme, the linked issues of not belonging and discrimination and racism framed the experience of ageing, and perceptions and experiences of dementia specifically. Our second theme, responding to dementia, provided the cultural context of dementia, addressing issues of knowledge and attitudes, service provision and service access. Quotations are presented verbatim by role (Community Leader = CL and Community Member = CM), participant number and self-identified ethnic group (SA = South Asian, BC = Black Caribbean and BA = Black African (see Table 1 for details).

### The migrant lifecourse

Twelve of our participants were first-generation migrants whilst the remainder were second generation. The migrant lifecourse provided the context for the experiences of ageing and later life within which people experienced dementia or memory problems. It is over 70 years since post-war migrants from the Caribbean first arrived in the UK in 1948. They were followed by others from the Indian sub-continent and Uganda until migration was curtailed from 1968 onwards. This group had a distinct lifecourse experiencing significant racism and discrimination in education, employment, and housing, all factors which contributed to the Race Relations Act of 1968 and subsequent wider equalities legislation. We identified two key sub-themes within the migrant lifecourse: not belonging, and discrimination and racism.

Participants talked about feeling far from home, looking back to the country that they (or their parents) migrated from, and described a sense of surprise that they had not returned home but rather aged in a foreign land. Underpinning this was a sense of not belonging, which was expressed in both emotional and physical ways. The emotional sense of not quite ‘belonging’, not being a full member of society and not feeling fully ‘at home’ was palpable: *“it marginalises you to such an extent, whereby you don’t feel you belong, so there’s issues of hostility and unwelcoming”* (CL1SA). This was developed further by another community leader, who had lived in the UK for 46 years: “*this is your home, we are as we came, that’s how I look at it. To me is that a lodger … a lodger come to your house, is paying to stay in your house, but ‘’it’s your home so you know every corner of your house”* (CL10BC). Although many participants may have come to the UK not intending to stay, they had since built their lives here, although the desire to return home was ever-present. As one Caribbean community leader explained, “*the people who are ageing here, they are ageing physically relatively well, but emotionally, these are the people who have failed to go home because originally, they had planned to come and go home… they have chosen to stay because their children are here now, and their children don’t want to go home because it is not their home”* (CL11SA). For some the desire to go home was not fulfilled until death, “*some people, when they came, they never really settled in their minds, emotionally, people always had that desire to return home*, *to be buried at home and things like that*” (CL12BC).

The physical and visual sense of not belonging was exemplified by a community leader, born in the UK during the 1950s, who had as a child felt that something was ‘wrong’ with her. Describing visiting her grandparents in Jamaica she observed: “*I always thought something was wrong because it’s only me and my brother that had my colour and when the ship pulled in Kingston Harbour, I realised nothing was wrong because I saw some children my colour (laughter) lighter and darker, I realised nothing was wrong, I do belong”* (CL5BC). In combination, this feeling of not belonging contributed to a perception among participants that they were not full members of society, with potential consequences for accessing age-related health care services.

Understanding and experiences of dementia and potential use of services were framed by a hostile environment reflecting the societal context of hostility, low expectations of minority groups and personal experiences of racism and discrimination. Participants had experienced an overtly hostile social environment where immigrants were ‘othered’: “*you have the national discourse of racism and discrimination and prejudice, xenophobia, dress code, Muslim hate, the negative perceptions that are instilled in the public conscience and then there’s also the pressure of being different and being the other”* (CL11SA). Accessing services and support for dementia may be more problematic when viewed through the prism of a less than welcoming environment, since “*it marginalises you to such an extent, whereby you don’t feel you belong, so there’s issues of hostility and unwelcoming”* (CL1SA).

Personal experiences of racism were recounted, such as “*I have experienced racism in every walk of life” and “... I can compare people that I went to school with so I know that no one can tell me that race and my colour does not affect my life”* (CM5BC). Others had strong memories of observing racism: “*in my early years I have to say I understood and if you get the drift here, I understood the metaphors of blacks, Irish and dogs, that was what was put on the doors –... which was telling you don’t knock here we don’t have any room for you. I understood it but I never really experienced it”* (CM2BC). Participants articulated the barriers to personal and professional achievement they faced: “*As a minority, you’re constantly having to prove yourself, that you’re worthy, that I’m worthy, that I’m capable of... I’m resigned to my fate and my fate tells me that glass ceiling is something we can look at too, but you can’t break through*” (CL1SA). These low expectations were recounted in the health context in terms of expectations for cognitive function: “*Very often, I’m afraid, the expectation of minority, black particularly, the expectation is very low and if they perform at a certain level, the comment will be made, oh that’s good for a black person*” (CL6BA). Similarly, another carer observed “... *expectations *[of cognitive ability] *can be lower for people of our ethnicity (black) when you’re telling people that there was a change, she’s finding things more difficult, in comparison to their expectation of what she should be doing, which is very low, then there was no issue *[for the health care staff]” (CM4BC).

### Responding to dementia

This theme encompassed knowledge and understanding of dementia, cultural appropriateness, and inequalities in care. Participants from both minority ethnic groups talked about lack of knowledge: “*I don’t think they* [the Asian community] *see memory problems as an issue… not many people know what dementia is, from my experience. They think it just comes with old age. They don’t actually take action or do anything about it”* (CL9SA). Linked to this were notions of denial and stigma: “*They’re very embarrassed. And many don’t want to admit it or maybe the children are not that observant to realise that something is amiss. But I think we try to … not to tell the world what’s happening, because we just think it’s something you deny I think”* (CL5BC). Such attitudes and the perceived stigma stopped people accessing services: *“I wouldn’t say the Black community so much but the Asian community, the minority community, don’t access services as they should, or as they could, simply because there’s a stigma attached to it”* (CL4SA). CL7SA developed this further: “*They don’t like confronting those kinds of things, you know, like cancer or mental health*. *There’s always quite a stigma about it rather than going out and seeking the hel*p. *They try and keep it contained and keep it secret then obviously that doesn’t really help anyone”*.

It was fully acknowledged that there are services to support people with dementia, but many are not culturally appropriate: “*There’s lots of services I know that are commissioned and that’s out there, but not exactly appropriate culturally or sensitive or in terms of the language, even, for people to access*” (CL2BC). CL4SA observed, “*What ends up happening is that these people who don’t speak the language, English, they don’t speak English clearly, they suffer the most because there is nothing available for them*”. Once services were accessed, the absence of culturally contextualised care was evident: “*It becomes much more important than any kind of normal functioning and support and if the person who is standing across you doesn’t understand the language, doesn’t understand what you’re talking about then it becomes a barrier*” (CL8SA). Failure to meet cultural needs in care homes and other settings has an impact on both on the person with dementia and their family: “*There is always the issue of the food and the skin care which you are still going to have to do yourself because the home can’t do that*” (CL12BC). We can surmise that this lack of personalisation is especially distressing for those from minority ethnic backgrounds given the importance of routine and familiarity in the context of daily life within a care setting for people with dementia.

Issues around culturally appropriate care were located within a broader narrative of unequal access to care, concerns about quality of care and lack of trust, which links back to the racism and discrimination sub-theme. One community leader, CL5BC, eloquently summed up the disadvantages that minority ethnic groups experienced accessing services: “*Yes, well to be honest, if you change yourself into a black person for a day, you will meet tremendous hardship, tremendous hardship, just by presenting yourself into any organisation or institution, you are ten steps behind your white counterpart”*. CL2BC further noted that, *“we will have to try doubly hard to get something of quality service”*. Once the system had been accessed, the perception of an unequal quality of care then came to the fore: “*I think sometimes maybe people might be not understood because people might have undergone all kinds of discrimination in their life and this may just be another set of experiences whereby, they feel not treated as the same as anybody else or are not getting the services like anybody else*. *You know, they don’t go because of this … they are going to be discriminated* [against]*”* (CL6BA). However, some participants noted that the care they received was because of the poor GP service: *“I didn’t feel that* [racism or discrimination was an issue]*, I just thought the doctor was bloody useless quite frankly, that’s what I thought she was quite useless”* (CM6BC), indicating that poor quality care was located within a broader framework of system failure.

## Discussion

In this paper we examined how dementia is understood and experienced by post-war minority ethnic people from the Caribbean and Indian sub-continent. We explored the understandings, experiences, and views of twelve community leaders, because of their acknowledged role supporting the understanding of dementia within their communities (Baghirathan et al., 2020) and six community members with lived experience of dementia. Across both groups our data encapsulate two themes. The migrant lifecourse theme highlighted the physical and emotional sense of not belonging, and the racism and discrimination which has characterised the experiences of this group. This theme illustrates how lifecourse experiences of negative and overtly hostile social and policy environments and services can be profound and long-lasting and provides a prism through which dementia and allied services are experienced. The second theme focused on the context of dementia care and highlighted dementia-specific issues of knowledge and understanding of dementia.

Findings from the cultural context of care theme – knowledge, stigma, and the cultural inappropriateness of dementia services – have been reported in other UK studies (Herat-Gunaratne et al., 2020; James et al., 2022). Some researchers contest the focus on lack of knowledge and understanding of dementia among minority ethnic groups (see Fletcher et al., 2022). However, for our participants this was seen as a genuine challenge that still needed to be acknowledged and addressed (Berwald et al., 2016; Blakemore et al., 2018). We are not seeking to problematise minority ethnic groups, but rather to problematise dementia services (Roberts et al., 2015; Fletcher et al., 2022). The findings argue for the inclusion of diverse views and lifecourse experiences within the context of developing a dementia strategy for research, policy and practice that is appropriate for a multicultural and heterogenous society (NHS National Health Service England, 2023; Alzheimer Europe, 2018; Parveen et al., 2018). The need for culturally appropriate care was located within a broader narrative of perceived inequities in access to, and quality of, care by minority ethnic groups.

It is important to acknowledge the limitations of this study which was undertaken in one specific area, London, and Southeast England. It is also important to acknowledge our positionality in that our interviewer, the wider authorship team and the participants were not ethnically matched (see Roche et al., 2021). Whilst recognising the importance of this argument, we suggest that the proposed binary insider/outsider relationship divide does not fully recognise the complexity of the researcher/research participant relationship which includes age, gender, education, and social class as well as ethnicity. Given the importance of religion and religious beliefs in previous research, we ensured the inclusion of faith leaders as well as third sector and statutory organisations. The COVID-19 pandemic precluded the completion of our recruitment strategy for those with lived experience of dementia. We fully accept that the perspectives of these two groups, people with dementia and their carers, are less visible because of this. We further acknowledge that, whilst we are focusing on ethnicity, there are other important characteristics such as gender and socio-economic status which influence attitudes towards, and experiences of, dementia. There is clearly considerable scope for research that adopts an intersectional approach towards experiences and understanding of dementia.

## Conclusion

The ageing in place of post-war migrant communities is bringing an element of cultural diversity to the UK older adult population which will become increasingly heterogeneous in coming decades. This will be reflected in the caseloads of the major age-related diseases, of which dementia is the exemplar. Families, communities, and service providers need to understand and respond appropriately to the growing importance of dementia as a health challenge for minority ethnic communities by developing a culturally informed policy, practice, and research agenda.

## Data Availability

The data that support the findings of this study are available from the corresponding author upon reasonable request.
